# Arterial dissection in childhood Takayasu Arteritis: not as rare as thought

**DOI:** 10.1186/s12969-016-0115-3

**Published:** 2016-09-22

**Authors:** Florence A. Aeschlimann, Lars Grosse-Wortmann, Susanne M. Benseler, Ronald M. Laxer, Diane Hebert, Rae S.M. Yeung

**Affiliations:** 1 Division of Rheumatology, Department of Paediatrics, The Hospital for Sick Children, University of Toronto, Toronto, ON Canada; 2 Division of Cardiology, Department of Paediatrics, The Hospital for Sick Children, University of Toronto, Toronto, ON Canada; 3 Rheumatology, Department of Paediatrics, Alberta Children’s Hospital, University of Calgary, Calgary, AB Canada; 4 Division of Nephrology, Department of Paediatrics, The Hospital for Sick Children, University of Toronto, Toronto, ON Canada; 5 Department of Immunology and Institute of Medical Science, University of Toronto, Toronto, ON Canada

**Keywords:** Takayasu arteritis, Vasculitis, Children, Imaging, Arterial dissection

## Abstract

**Background:**

Arterial vessel wall dissection is a rare, life-threatening and rarely described complication in childhood Takayasu Arteritis (cTA). Prevalence and risk factors for arterial dissection in cTA are unknown. We sought to study the prevalence and analyse risk factors for arterial dissection in cTA.

**Findings:**

A single center retrospective review of all children with cTA was performed. Patients with arterial dissection at cTA diagnosis were reported in detail and compared to the remaining single center retrospective cohort of children without dissection. Disease activity was assessed by the Pediatric Vasculitis Disease Activity Score (PVAS). A total of 27 cTA patients (74 % girls) were included. Three children (11 %) presented with dissection at diagnosis of cTA. They had higher PVAS (median 21 versus 10, *p* = 0.26), increased neutrophils (*p* < 0.0001) and lower albumin levels (*p* = 0.05). Arterial hypertension was common in both groups: in 67 % of children with dissection and 54 % of those without.

**Conclusions:**

Arterial dissection was more frequent in our cTA cohort than previously reported. Careful vascular imaging assessment is crucial to document this complication. High disease activity and markers of inflammation especially in combination with arterial hypertension, may be associated with the risk for vessel wall dissection in children with cTA.

## Background

Childhood Takayasu Arteritis (cTA) is a devastating vasculitis characterised by granulomatous inflammation of the aorta and its major branches resulting in severe ischemic organ dysfunction [1]. Arterial dissection is a rare, life-threatening complication [2]. The prevalence of and risk factors for dissection in cTA are unknown. Therefore, the aims of this study were to: 1) determine the prevalence of dissection in cTA; 2) compare clinical, laboratory and imaging characteristics of cTA with and without dissection and 3) identify risk factors for dissection in cTA.

## Findings

### Hypothesis

Childhood TA patients with dissection have higher disease activity compared to cTA patients without dissection.

### Methods

This single, tertiary care center study included all consecutive children diagnosed with cTA and meeting the EULAR/PRINTO/PReS classification criteria between 1986 and 2015 [3]. Before 2009, all children were diagnosed based on the American College of Rheumatology criteria and also met the EULAR/PRINTO/PReS classification criteria for cTA [4, 5]. Children were identified from the institutional databases; data were captured using standardized forms under an Institutional Research Ethics Board approved study which waived the requirement for informed consent.

Demographic, clinical, laboratory and imaging data at presentation were captured. Disease activity was determined using the validated Pediatric Vasculitis Activity Score (PVAS) [6]. Arterial dissection was defined as presence of a double lumen with visible intimal flap and/or an intramural hematoma on vascular imaging [7]. Magnetic resonance imaging with angiography (MRI/A) has become the first line imaging modality in cTA in our institution due to its reduced exposure to ionizing radiation and its non-invasive procedure when compared to conventional angiography. All images were reviewed by an expert reader in cardiovascular imaging with more than 10 years experience (LGW) in addition to standard clinical review as part of patient management. Groups were compared using parametric and non-parametric tests as appropriate; analyses were conducted in Prism (GraphPad 6.0 g, San Diego).

### Results

**Fig. 1:**
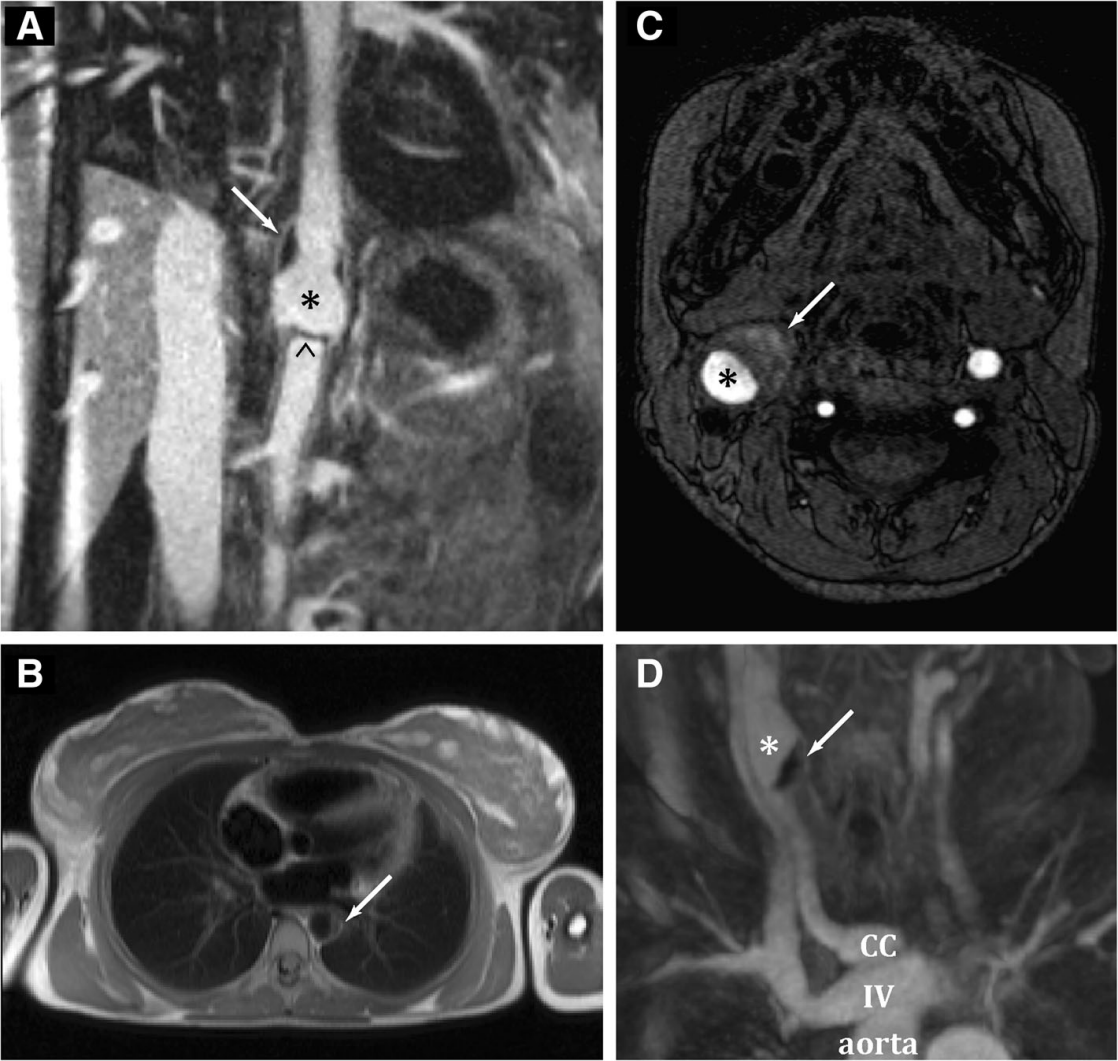
Vascular imaging in the three children with Takayasu arteritis and dissection. Legend: Patient 1: **a** A coronal reformat of the 3D volume rendered MRA dataset demonstrates the intramural hematoma (arrow) as well as a dissection flap (arrowhead) at the caudal end of the aneurysm (*) in the abdominal aorta. The intramural hematoma (arrow) is located at the proximal end of the aneurysm. The celiac axis arises immediately distal to the dissection flap with a stenotic origin. There is thickening of the aortic wall and a long-segment narrowing of the infra-renal aorta. Patient 2: **b** A T2 weighted turbo spin echo magnetic resonance image in the axial orientation shows a dissection flap in the thoracic descending aorta at the level of the left atrium (arrow). Patient 3: **c** The T1 weighted turbo spin echo and **d** the para-coronal reformat of a contrast-enhanced magnetic resonance angiogram show an aneurysm of the right carotid artery (*) with intramural hematoma (arrow). CC = Common carotid artery, IV = innominate vein

A total of 27 cTA patients (74 % female) were included. Median age at diagnosis was 12.4 years (range 4.2–17.7). Spontaneous dissection was found in 3/27 children (11 %). Dissections were seen in the abdominal aorta in two and in the carotid artery in one child. No additional dissections were detected during the follow-up period. The three children and their vascular findings are described below, and detailed in Table 1 and Fig. 1.

**Table 1:** Characteristic features of the patients with cTA and dissection

	Patient 1	Patient 2	Patient 3
Age at diagnosis	14.6 years	12.4 years	10.3 years
Age at symptom onset	14.4 years	?	10.0 years
Gender	Female	Female	Female
Ethnicity	First Nations	African	East Indian
Presenting symptoms	Constitutional symptoms, abdominal pain, headache, cough, chest pain, dyspnea	Constitutional symptoms, bilateral cervical lymphadenopathy	Constitutional symptoms, vomiting, left focal seizures with secondary generalization
Clinical findings	Arterial hypertension, weak femoral and pedal pulses, BP discrepancy of 50 mmHg, cardio-respiratory failure	Decreased left radial and peripheral pedal pulses	Arterial hypertension, midline abdominal bruits, BP discrepancy, pulsatile right cervical mass
Laboratory findings	WBC 13.6 × 10^9^/L Neutrophils 12.3 × 10^9^/L Hb 83 g/l ESR 31 mm/h CRP 14.5 mg/dL Albumin 25 g/L Creatinine 123 umol/l	WBC 5.17 × 10^9^/L Neutrophils not done Hb 144 g/l ESR 32 mm/h CRP not done Albumin 36 g/L	WBC 22.3 × 10^9^/L Neutrophils 19.1 × 10^9^/L Hb 115 g/l ESR 40 mm/h CRP 24.2 mg/dL Albumin 36 g/L
Radiological findings	CTA and MRI/A: Thickening of entire abdominal aorta with dilatation and post-contrast enhancement of the wall. Dissection of the abdominal aorta with intramural hematoma. Severe narrowing of SMA, celiac artery and both renal arteries.	MRI/A: Extensive arteritis involving aortic arch, entire descending aorta and upper abdominal aorta with lumen irregularity, vessel wall thickening and post-contrast enhancement. Descending aorta with two areas of dilatation, in between narrowing with evidence of dissection. L subclavian artery severely narrowed.	MRI/A: Thickening and post-contrast enhancement of the abdominal aorta with evidence of an aneurysm. R carotid artery aneurysm with perivascular edema and vessel inflammation, intramural hematoma and suspected dissection flap. Bilateral renal artery stenosis; dilatation of the origin of the celiac trunk and SMA.
PVAS (max. score 63)	21	8	25
Treatment	Induction: Pulse Methylprednisolone, then high-dose Prednisone Methotrexate orally Infliximab every 4 weeks	Induction: High dose Prednisone	Induction: High-dose Prednisone Methotrexate subcutaneously
	Other: Quadruple anti-hypertensive therapy ASA	Other: Anti-tuberculosis therapy	Other: Quintuple anti-hypertensive therapy ASA
Follow-up duration	26 months	6 years	36 months

*Legend*: *BP discrepancy* blood pressure (BP) discrepancy of >10 mmHg between any limb, *PVAS* pediatric vasculitis activity score, *WBC* white blood cell count, *Hb* hemoglobin, *ESR* erythrocyte sedimentation rate, *CRP* C-reactive protein, *CTA* computer tomography angiography, *MRI*/*A* magnetic resonance imaging/angiography, *SMA* superior mesenteric artery, *R* right, *L* left, *ASA* acetylsalicylic acid

#### Patient 1

A 14-year-old girl was referred with a two-month history of constitutional symptoms. She presented with acute hypertensive crisis and cardiovascular decompensation. She had weak femoral pulses and blood pressure discrepancy between limbs. MRI/A was consistent with cTA and revealed a large dissection flap in the abdominal aorta (Fig. 1a). Quadruple antihypertensive treatment and pulse methylprednisolone were started. While attempting to lower the blood pressure, the renal perfusion deteriorated resulting in renal failure. An emergent axillary-femoral artery bypass conduit was placed to provide retrograde renal perfusion resulting in rapid improvement of renal function and blood pressure control. The aortic aneurysm and the dissection flap were left unchanged. Subsequently, methotrexate and infliximab were started and prednisone was tapered. She achieved remission at six months of therapy and continued to be in remission on medication including methotrexate, infliximab and low-dose prednisone at 26 months of follow-up. Repeat MRI/As showed no evidence of new vascular lesions or ongoing vessel inflammation. While the dissection flap remained unchanged during initial follow-up imaging, the last MRI/A at 26 months of follow-up showed a spontaneous reposition of the dissection flap along the aortic wall.

#### Patient 2

A 12-year-old girl presented at a local hospital with constitutional symptoms and bilateral cervical lymphadenopathy. She was normotensive, but had decreased left radial and pedal pulses. Lymph node biopsy showed active extrapulmonary tuberculosis (diagnosis confirmed by positive culture); clinically and radiologically there was no evidence of tuberculosis infection in the lung or any other anatomical site. Quadruple anti-tuberculosis treatment was started. MRI/A demonstrated extensive aortitis with a dissection in the abdominal aorta (Fig. 1b). Following diagnosis of cTA high-dose prednisone was initiated. Due to active vessel wall inflammation surgical intervention was not considered a therapeutic option. Repeat imaging at six months revealed radiographic progression, despite a clinically stable course. Azathioprine was added, but inflammatory markers increased further. Azathioprine was switched to mycophenolate mofetil due to persistent leucopenia. She was referred for second opinion two years after diagnosis. Investigations revealed increased inflammatory markers. MRI/A showed vessel wall thickening and persistent aortic dissection. There was no evidence of ongoing tuberculosis infection. Treatment modifications including increased prednisone were suggested. She discontinued the treatment three years after diagnosis against medical advice. Regular follow-up MRI/As were performed, the last one, 6 years after diagnosis, did not show evidence of active disease or dissection; clinically she was asymptomatic.

#### Patient 3

A 10-year-old girl presented with three episodes of focal seizures with secondary generalization. She had a three-month history of constitutional symptoms. Clinical examination revealed severe hypertension, midline abdominal bruits and a right cervical pulsatile mass. Brain MR was consistent with posterior reversible encephalopathy secondary to arterial hypertension. MRI/A findings were consistent with cTA, an intramural hematoma with suspected dissection flap was seen in the right carotid artery (Fig. 1c and 1d). She was started on high-dose prednisone, methotrexate and quintuple antihypertensive treatment. The intramural hematoma with suspected dissection flap was managed with conventional treatment, as active vessel wall inflammation precluded a surgical intervention. While tapering prednisone, inflammatory markers increased. Repeat MRI/A at eight months revealed new aortic lesions and an increasing carotid aneurysm. Tocilizumab was added. At 36-month follow-up, she continued to be asymptomatic with normal inflammatory markers while treated with tocilizumab, methotrexate and low-dose prednisone. Repeat MRI/A revealed no evidence of active disease; the right carotid artery hematoma had resolved; a dissection flap was not detected. The carotid artery aneurysm remained unchanged.

In all three patients with dissection, potential other causes of arterial dissection such as syphilis and HIV were excluded serologically. All children were tested for tuberculosis infection at diagnosis of cTA. One child in the dissection group (1/3, 33 %) and 2 children in the non-dissection group (2/24, 8 %) were diagnosed with tuberculosis infection. Patients with dissection (*N* = 3) and those without (*N* = 24) are detailed in Table 2. At diagnosis, there was no statistically significant difference in gender, age, presence of specific clinical symptoms or tuberculosis infection when comparing cTA patients with dissection and those without. Hypertension was common, with 67 % being hypertensive in the dissection group compared to 54 % in the non-dissection group (*p* = 1). Overall, children with dissection had a trend towards higher disease activity at diagnosis as measured by median PVAS (21 versus 10), but this was not statistically significant (*p* = 0.26). They had significantly higher neutrophil counts (mean 15.7 × 10^9^/L versus 5.8 × 10^9^/L, *p* < 0.0001) and lower albumin levels (mean 32.3 g/l versus 41.1 g/l, *p* = 0.05). There was no statistically significant difference seen in ESR, hemoglobin, platelets, creatinine and von Willebrand factor antigen levels.

**Table 2:** Demographics, clinical and laboratory features in cTA patients with and without dissection

	cTA with dissection (N = 3)	cTA without dissection (N = 24)	*P*-value
Age at diagnosis in years, mean (SD)	12.4 (2.2)	11.5 (3.7)	0.37
Female (%)	3 (100)	17 (71)	0.55
Tuberculosis (%)	1 (33)	2 (8)	0.29
Arterial hypertension (%)	2 (66)	13 (54)	1
Constitutional symptoms (%)	3 (100)	14 (58)	0.27
PVAS, median (IQR)	21 (8–25)	10 (6–35)	0.26
ESR in mm/h, median (IQR)	32 (31–40)	35 (1–109)	0.89
Albumin in g/L, mean (SD)	32.3 (6.4)	41.1 (6.6)	0.05
Hemoglobin in g/L, mean (SD)	113.7 (31.0)	112.8 (18.4)	0.95
WBC in x10^9^, mean (SD)	13.7 (8.6)	9.3 (3.0)	0.07
Neutrophils in x10^9^, mean (SD)	15.7 (4.8)	5.8 (2.3)	<0.0001
Platelets in x10^9^, mean (SD)	349 (183)	401 (155)	0.60
Imaging at diagnosis (%)			
MRI/A	3 (100)	5 (21)	0.02
Conventional angiography	0	6 (25)	1
Combined (MRI/A, CTA, angiography)	0	13 (54)	0.22

*Legend*: Data are presented as counts with percentages, mean with standard deviation (SD) or median with interquartile range (IQR). Constitutional symptoms included malaise, weight loss, fever and lymphadenopathy. C-reactive protein was not included into the analysis, as the assay method changed during the study period

*PVAS* pediatric vasculitis activity score, *ESR* erythrocyte sedimentation rate, *WBC* white blood cell count, *MRI*/*A* magnetic resonance imaging with angiography, *CTA* computer tomography angiography

## Discussion

This is the first study to determine the prevalence of dissections in cTA; one in 10 children with cTA (3/27, 11 %), had evidence of dissection on initial imaging. To date, only one case report of dissection in a child with cTA has been published [2]. Interestingly, large cTA cohorts have not reported dissection at diagnosis, including those providing detailed description of vessel wall disease [8–12]. Overall, improved vessel wall imaging techniques have resulted in more detailed characterization of the vascular structure [13]. Dissection has important implications for therapeutic decisions, especially involving anti-coagulation and anti-platelet agents. In the presence of an intramural hematoma anti-coagulation should be avoided due to increased bleeding risk.

This study analysed possible risk factors for dissections in cTA. Children with dissection tended to present with evidence of high disease activity including high PVAS, increased neutrophils and lower albumin levels. Disease activity and inflammation in Takayasu Arteritis (TA) is thought to be mediated through activated monocytes, macrophages and T-cells that synthetize pro-inflammatory cytokines, including TNF-alpha and IL-6 [14]. These cytokines in particular mediate production of matrix metalloproteases (MMPs), a family of proteolytic enzymes able to destroy components of the extracellular matrix – the scaffolding holding the vessel wall components together. In adults with TA, higher levels of MMPs, especially MMP-9, and lower levels of MMP inhibitors correlate with disease activity [15, 16]. Further, distinct MMP subtypes have been associated with aneurysms and dissection in other vascular diseases including Kawasaki disease and type A acute aortic dissection [17, 18]. In general, children present with hyperacute inflammation at diagnosis of TA, which was also seen in our patient population. This may put them at higher risk for dissection, despite a lack of statistical difference in surrogate measures of inflammation in our very small cohort. Interestingly, one child presented with concomitant Mycobacterium tuberculosis infection. In tuberculosis, intercellular networks including TNF-alpha also drive increased MMP gene expression and secretion [19].

Even though the presence of arterial hypertension was not statistically significantly different in the dissection group, it may play an important role in the development of dissection. Hypertension-related rupture of the vasa vasorum with subsequent development of an intramural hematoma has been suggested as possible pathomechanism for dissection in acute aortic syndromes in adults [20]. The vasa vasorum feed the media layer of vessels and their rupture may lead to creation of an intramural hematoma with or without intimal tear [20]. This mechanism is even more deleterious in vessels with wall inflammation and potential distal stenosis as demonstrated in our first patient. This may create critical pressure in a weakened vessel wall and subsequently increases the risk for dissection.

There are several limitations to the study. This is a retrospective study spanning three decades and different therapeutic management regimens. The cohort was followed at a single tertiary care center, which may result in inclusion of sicker children and overestimation of the prevalence of dissections. In contrast, vascular imaging techniques have dramatically improved, which may have led to underestimation of dissections in those diagnosed earlier. The patient numbers are small and results may not be representative, but nonetheless, highlight the importance of this complication, as dissection has important implications for therapeutic decisions, especially involving anti-coagulation and anti-platelet agents. Further studies are necessary to confirm our preliminary data.

## Conclusion

Dissection in cTA appears to be more frequent than previously appreciated. Careful vessel wall assessment with high quality vascular imaging is therefore required in these patients. In this study, statistically significant difference between children with dissection and those without dissection was not found, most likely due to the small sample size. Nonetheless, high disease activity and inflammation especially in combination with hypertension, may increase the risk for development of dissection in children with TA. Recognition and identification of these risk factors are important in the management of cTA.

## Abbreviations

ASA: Acetylsalicylic acid
BP discrepancy: Blood pressure discrepancy
CC: Common carotid artery
CRP: C-reactive protein
cTA: Childhood Takayasu Arteritis
CTA: Computer tomography angiography
ESR: Erythrocyte sedimentation rate
EULAR/PRINTO/PReS: European League against Rheumatism (EULAR)/Pediatric Rheumatology International Trials Organization (PRINTO)/Pediatric Rheumatology European Society (PReS)
Hb: Hemoglobin
IV: Innominate vein
MMP: Matrix metalloprotease
MRI/A: Magnetic resonance imaging/angiography
PVAS: Pediatric vasculitis activity score
SMA: Superior mesenteric artery
WBC: White blood cell count
